# *Cryptococcus neoformans* Escape From *Dictyostelium* Amoeba by Both WASH-Mediated Constitutive Exocytosis and Vomocytosis

**DOI:** 10.3389/fcimb.2018.00108

**Published:** 2018-04-09

**Authors:** Rhys A. Watkins, Alexandre Andrews, Charlotte Wynn, Caroline Barisch, Jason S. King, Simon A. Johnston

**Affiliations:** ^1^Bateson Centre, University of Sheffield, Sheffield, United Kingdom; ^2^Department of Infection Immunity and Cardiovascular Disease, Medical School, University of Sheffield, Sheffield, United Kingdom; ^3^Department of Biochemistry, Faculty of Science, University of Geneva, Geneva, Switzerland; ^4^Department of Biomedical Sciences, University of Sheffield, Sheffield, United Kingdom

**Keywords:** cryptococcus, *Dictyostelium*, amoeba, pathogen, exocytosis, cryptococcosis, vomocytosis, WASH

## Abstract

*Cryptococcus neoformans* is an environmental yeast that can cause opportunistic infections in humans. As infecting animals does not form part of its normal life-cycle, it has been proposed that the virulence traits that allow cryptococci to resist immune cells were selected through interactions with environmental phagocytes such as amoebae. Here, we investigate the interactions between *C. neoformans* and the social amoeba *Dictyostelium discoideum*. We show that like macrophages, *D. discoideum* is unable to kill *C. neoformans* upon phagocytosis. Despite this, we find that the yeast pass through the amoebae with an apparently normal phagocytic transit and are released alive by constitutive exocytosis after ~80 min. This is the canonical pathway in amoebae, used to dispose of indigestible material after nutrient extraction. Surprisingly however, we show that upon either genetic or pharmacological blockage of constitutive exocytosis, *C. neoformans* still escape from *D. discoideum* by a secondary mechanism. We demonstrate that constitutive exocytosis-independent egress is stochastic and actin-independent. This strongly resembles the non-lytic release of cryptococci by vomocytosis from macrophages, which do not perform constitutive exocytosis and normally retain phagocytosed material. Our data indicate that vomocytosis is functionally redundant for escape from amoebae, which thus may not be the primary driver for its evolutionary selection. Nonetheless, we show that vomocytosis of *C. neoformans* is mechanistically conserved in hosts ranging from amoebae to man, providing new avenues to understand this poorly-understood but important virulence mechanism.

## Introduction

*Cryptococcus neoformans* is a basidiomycete yeast found globally in a wide variety of natural environments. Unusually for an environmental yeast, *C. neoformans* is also a pathogen of animals. Most significant is the fatal infection of the severely immunocompromised, with cryptococcal meningitis caused by *C. neoformans* responsible for 15% of AIDS-related deaths (Rajasingham et al., 2017). The interaction of *C. neoformans* with its host is highly complex, and what differentiates normal immunity from the development of life threatening cryptococcal meningitis is well defined (Tenforde et al., 2017).

Macrophages have been repeatedly demonstrated to be critical for protection against *C. neoformans* infection. However, macrophages may also have a role in pathogenesis in the immunocompromised as cryptococci are able to grow and survive within macrophages and may use macrophages as a Trojan horse to disseminate from the lung. *In vitro*, almost every aspect of macrophage antimicrobial activity is either avoided or manipulated by *C. neoformans*, which are able to survive and replicate intracellularly, following uptake by phagocytosis (Johnston and May, 2013; Ballou and Johnston, 2017). Survival traits include the generation of a characteristic polysaccharide capsule, which is both anti-phagocytic and helps protect the yeast from the host antimicrobial machinery if it is engulfed, as well as melanin production which serves as a potent antioxidant—protecting the yeast from the phagosomal oxidative attack and the immunomodulatory activity of cell wall chitin (Casadevall et al., 2000; Nosanchuk and Casadevall, 2006; Wiesner et al., 2015).

An additional pathogenic mechanism is the remarkable ability of *C. neoformans* to promote its non-lytic expulsion from host cells in a process known as vomocytosis (Alvarez and Casadevall, 2006; Ma et al., 2006). This enables the yeast to escape whilst leaving the host phagocyte intact, thus preventing immune stimulation and promoting dissemination. Whilst it has been shown that vomocytosis is suppressed by host actin polymerization (Johnston and May, 2010) and can be modulated by host Annexin A2 and Mitogen Activated Protein kinase (ERK5) activity (Stukes et al., 2016; Gilbert et al., 2017) little is known of the underlying molecular mechanisms underlying expulsion. Nonetheless, vomocytosis has been observed in both cell culture and *in vivo* models and is thought to significantly contribute to *C. neoformans* virulence (Alvarez and Casadevall, 2006; Ma et al., 2006; Bojarczuk et al., 2016; Johnston et al., 2016; Gilbert et al., 2017).

As with other opportunistic pathogens, it is unlikely that interactions with mammalian macrophages have been the evolutionary drivers of *C. neoformans* virulence. Cryptococci are free-living fungi with a life cycle that is not dependent on infecting an animal host. It has therefore been proposed that the mechanisms that allow *C. neoformans* to survive and grow in macrophages have primarily evolved to avoid predation by phagocytes in its natural environment, such as amoebae (Steenbergen et al., 2001; Casadevall, 2012; Watkins et al., 2017).

Like leukocytes, amoebae are professional phagocytes, using their chemotactic and phagocytic abilities to capture and kill environmental microbes for food. Despite the large evolutionary distance between them, much of the machinery and mechanisms for phagocytosis and phagosome maturation are highly conserved between amoebae and mammalian immune cells (Boulais et al., 2010). Traits that have evolved to help yeast and bacteria avoid being killed by amoebae in the environment are therefore likely to have similar effects when they encounter mammalian immune cells.

Previous studies have demonstrated similarities in the interactions between *C. neoformans* with amoebae and macrophages. *C. neoformans* is able to both survive phagocytosis and replicate intracellularly within *Acanthamoeba castellanii*, ultimately being released alive without causing lysis of the host amoeba (Steenbergen et al., 2001; Chrisman et al., 2010). Due to its amenability to genetic manipulation, the social amoeba *Dictyostelium discoideum* has been used a model host for a number of human pathogens and is also susceptible to *C. neoformans* infection (Steenbergen et al., 2003). Importantly, passage through *D. discoideum* caused a stimulation in *C. neoformans* capsule expansion and melanization together with a corresponding increase in subsequent virulence in mice (Steenbergen et al., 2003). Interactions with amoebae can therefore directly influence interactions between *C. neoformans* and mammalian immune cells.

The fate of internalized material in animal cells is variable and complex. There are examples of the expulsion of internalized material from a variety of cell types, particularly in the context of antigen presentation (Chen and Jondal, 2004; Peters et al., 2006; Griffiths et al., 2012; Le Roux et al., 2012; Turner et al., 2016). However, animal macrophages (notably tissue resident cells, such as alveolar macrophage) have the ability to retain particulate matter that may otherwise be damaging (Bai et al., 2015). In contrast, the constitutive exocytosis of phagocytosed material by amoeba has been demonstrated in diverse species including *D. discoideum, Amoeba proteus, Entamoeba histolytica* and *A. castellanii* (Weisman and Korn, 1967; Ravdin et al., 1988; Christofidou-Solomidou and Stockem, 1992; Clarke et al., 2010) and thus appears to be a necessary and general feature of free-living amoebae. Therefore macrophages and amoebae differ in their retention of phagocytosed material.

Recently it was shown that constitutive exocytosis in *D. discoideum* is dependent on the activity of the WASH (WASP And SCAR Homolog) complex (Carnell et al., 2011). WASH is a direct activator of the ARP2/3 (Actin Related Protein 2/3) complex, causing the polymerization of actin on the surface of vesicles and driving membrane protein sorting and recycling (Derivery et al., 2009; Gomez and Billadeau, 2009; Zech et al., 2011; Seaman et al., 2013). Whilst an early phase of WASH activity drives the retrieval of cell surface proteins from phagosomes (Buckley et al., 2016), a second phase of activity occurs after 40–60 min of digestion, driving the removal of the vacuolar (V)-ATPase and phagosomal neutralization. This facilitates hydrolase retrieval (King et al., 2013) and is a prerequisite for exocytosis. Consequently *D. discoideum* cells lacking WASH have a complete block in constitutive exocytosis (Carnell et al., 2011).

As constitutive exocytosis represents the normal mechanism of non-lytic release of phagosomal contents, we hypothesized that this may be involved in the escape of live *C. neoformans* from amoebae. We show that *C. neoformans* survive phagocytosis by *D. discoideum*, but follow an apparently normal phagosomal transit and are normally released alive by WASH-dependent constitutive exocytosis. However, when constitutive exocytosis is blocked, *C. neoformans* still escape in a WASH and actin-independent manner reminiscent of vomocytosis. This demonstrates redundant, mechanistically different egress mechanisms with implications for the understanding of the evolutionary drivers of cryptococcal virulence.

## Materials and methods

### Strains and cell culture

For all experiments the Ax2 axenic strain of *D. discoideum* was used, both as “wild type” as well as the parental of the previously published *WASH*-mutant strain (Carnell et al., 2011). *D. discoideum* were cultured in filter-sterilized HL-5 medium (Formedium, Norfolk, UK) at 22°C. Cells expressing GFP fused to the vatM subunit of the vacuolar-ATPase were generated using plasmid pMJC25 (Carnell et al., 2011).

Unless otherwise stated, *C. neoformans* var. grubii *(*serotype A) strain H99α stably expressing mCherry was used (Gibson et al., in review). The previously published *plb1* and *cap59* mutants were generated from the alternative wild-type parent H99, which was used as control when appropriate (Chen et al., 2000; Voelz et al., 2010). *C. neoformans* were grown in YPD medium at 28°C prior to experiments, but washed and resuspended in HL5 medium before infecting *D. discoideum*. *C. neoformans* were heat-killed by incubation at 65°C for 30 min, before washing in PBS. UV-killing was performed by exposure of *C. neoformans* cultures to 4 J of UV using a UVIlink CL-5087 cross-linker illuminator. Killing was always confirmed by plating samples on YPD agar to check absence of growth.

### Infections and microscopy

Prior to imaging, 2 × 10^6^ *D. discoideum* cells were seeded in 2 ml HL5 medium in glass-bottom 35 mm dishes (Mat-Tek), left to adhere for 30 min. Then 2 × 10^5^ *C. neoformans* cells, 4.5 μm green fluorescent YG unmodified beads (Polysciences Inc., Pennsylvania, USA), or TRITC-labeled heat killed *S. cerevisiae* (kind gift from Thierry Soldati; University of Geneva) were added prior to imaging. This differed for latrunculin treatment experiments: amoebae were mixed with yeast at a ratio of 1:1 and left for 1 h for phagocytosis to occur before addition of 5 μm Latrunculin A (Cayman chemical Co.).

Long term time-lapse movies were recorded using a Nikon TI- E with a CFI Plan Apochromat λ 20x N.A.0.75 at 22°C. Images were captured on a large-format Andor Neo 5.5 s CMOS camera for 12 h, imaging 4 fields of view every 30 s. Transit times were determined as the time from phagocytosis to release. Events where cells could not be tracked for a minimum of 500 min post-phagocytosis were excluded from the analysis.

Spinning disc microscopy was performed on a Perkin-Elmer Ultraview VoX spinning disk confocal microscope running on an Olympus 1 × 81 body with an UplanSApo 60x oil immersion objective (NA 1.4). Images were captured on a Hamamatsu C9100-50 EM-CCD camera.

### Statistical analysis

Mann Whitney test was used to test significance between continuous data and Fisher's exact test for categorical data. *P*-values below 0.05 (with modification for multiplicity of testing) were considered statistically significant. All statistical tests were performed with GraphPad Prism version 7.

## Results

### *Cryptococcus neoformans* is constitutively exocytosed by amoebae

To determine whether *C. neoformans* utilizes a pathogen-specific mechanism of release or follows the normal phagocytic pathway of engulfment and constitutive exocytosis in amoeba we first compared the transit of *C. neoformans* with that of inert particles.

**Figure 1:**
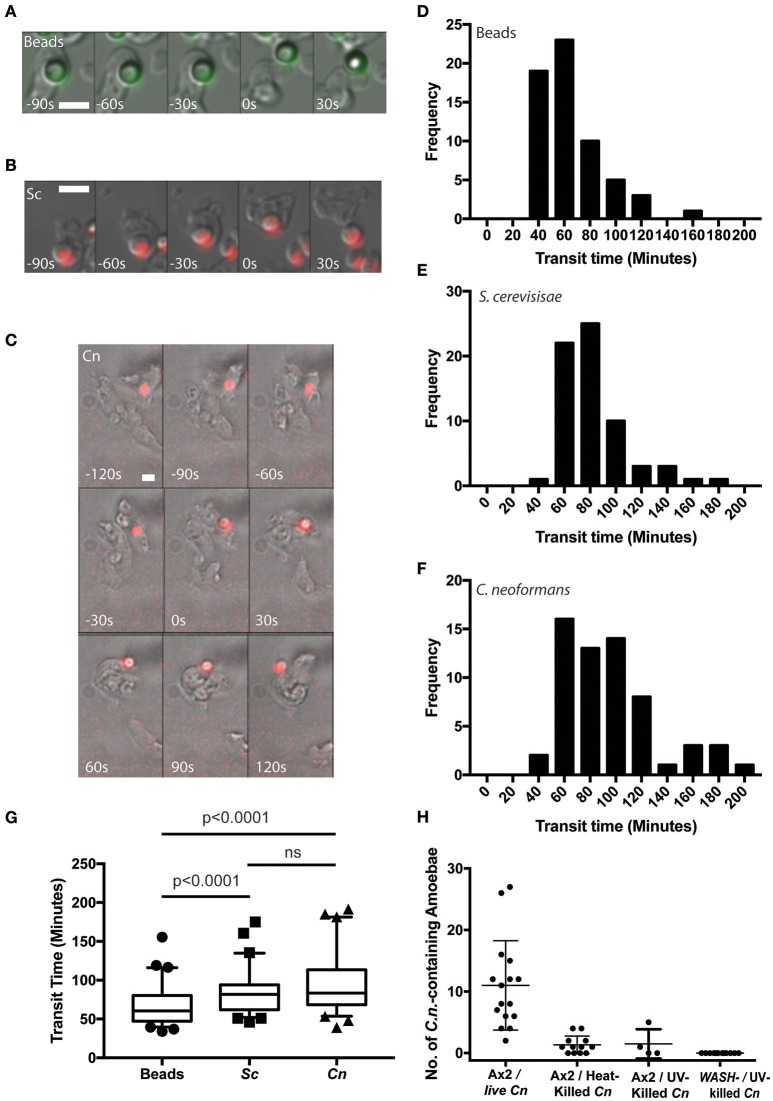
*Cryptococcus neoformans* is constitutively exocytosed from *Dictyostelium discoideum* amoeba. **(A–C)** Example exocytosis of **(A)** 4.5 um green fluorescent latex beads (images start at 40 min after phagocytosis) **(B)** heat killed *Saccharomyces cerevisiae* (images start at 60 min after phagocytosis) and **(C)** *C. neoformans* strain Kn99mCherry from wild type *D. discoideum* strain Ax2 (images start at 60 min after phagocytosis). Time 0 s indicates point of exocytosis. Scale bars 5 μm. (**D–F)** Frequency histograms of combined transit times measured from three independent 12 h time lapses. **(D)** Latex beads 126 transit times. **(E)** Heat killed *S. cerevisiae* 66 transit times. **(F)** *C. neoformans* 57 transit times. **(G)** Comparison of transit times for latex beads, heat killed *S. cerevisiae* and *C. neoformans*. **(H)** Quantification of phagocytic events for live, heat killed and UV killed cryptococci in Ax2 and WASH null cells. *P*-values are Mann-Whitney test.

Using time lapse microscopy, we followed the phagocytosis and release of *C. neoformans* compared to heat killed non-pathogenic yeast *Saccharomyces cerevisiae* and 4.0 μm latex beads by *D. discoideum*. All three cargoes were of a similar size (between 3 and 5 μm) and all were exocytosed from amoebae (Figures 1A–C). However, unlike the vomocytosis of *C. neoformans* from macrophages which happens stochastically and inefficiently over a period of many hours (Ma et al., 2006; Johnston and May, 2010), 100% of phagosomes fused with the plasma membrane, exocytosing their contents within 4 h of engulfment in all cases.

Surprisingly, whilst we attempted to use killed *C. neoformans* as controls, both heat- or UV-killing of the yeast reduced phagocytosis by *D. discoideum* by 90% (Figure 1H). This effect was further compounded in *WASH*-null amoebae, in which phagocytosis of UV-killed *C. neoformans* was never observed in 3 independent experiments, consistent with previous reports of phagocytosis defects in these mutants due to reduced surface levels of phagocytic receptors (Buckley et al., 2016). The reason why killed *C. neoformans* resist phagocytosis is unclear, but it was not possible to observe sufficient events for analysis and therefore heat-killed *S. cerevisiae* were used as non-pathogenic controls in subsequent experiments.

From the time-lapse movies, we were able to define precise transit times for phagosomes containing the different cargoes, tracking individual particles from engulfment to release (Figures 1D–F). Comparison of the transit time demonstrated that both fungal cells took slightly longer to complete the phagocytic cycle than latex beads however, there was no significant difference between heat killed *S. cerevisiae* and *C. neoformans* (median transit time latex beads = 61 min, heat killed *S. cerevisiae* = 82 min and *C. neoformans* = 84 min, Figure 1G). Importantly the *C. neoformans* were able to resist killing by the amoebae and were exocytosed alive, as indicated by subsequent budding and dividing after egress consistent with previous studies (Steenbergen et al., 2003). Therefore, despite their ability to survive phagocytosis by *D. discoideum*, transit time of *C. neoformans* is indistinguishable from normal constitutive exocytosis.

### The V-ATPase is rapidly recruited to phagosomes containing *C. neoformans* and removed prior to exocytosis

Unlike the vomocytosis of cryptococci, a number of molecular requirements for constitutive exocytosis in amoebae have been identified. We therefore next asked whether passage of *C. neoformans* through *D. discoideum* followed the normal path of maturation and constitutive exocytosis.

**Figure 2:**
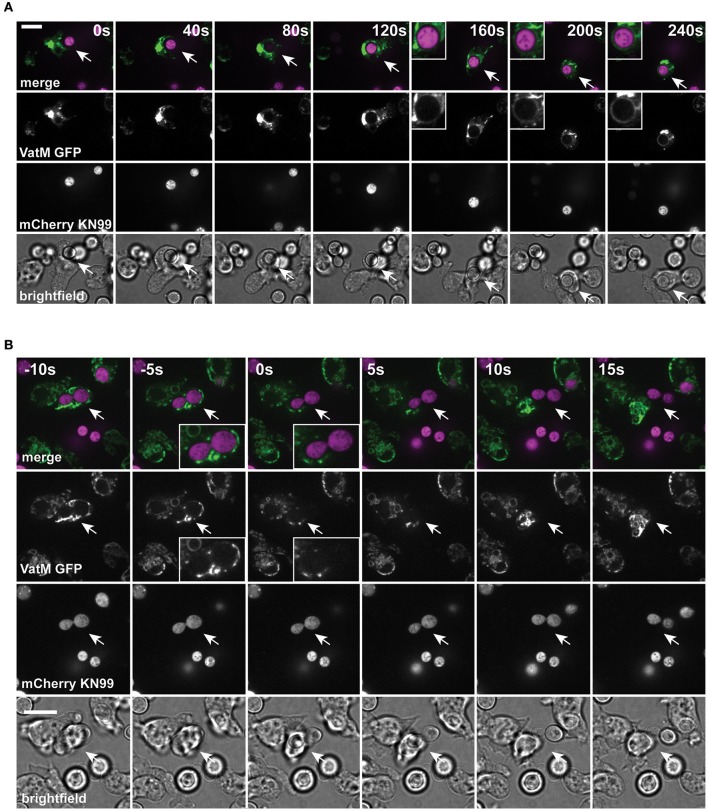
Phagosomes containing *Cryptococcus neoformans* acquire V-ATPase following phagocytosis that is lost prior to exocytosis. **(A)** Confocal time lapse microscopy of *C. neoformans* phagocytosis by wild type *D. discoideum* strain Ax2. Representative time lapse from three independent experiments. Images were captured every 10 s. VatM is a subunit of the *D. discoideum* V-ATPase complex. Arrow indicates phagocytosed cryptococcal cell. Inset box is a magnification of phagosome containing cryptococcal cell demonstrating acquisition of V-ATPase. **(B)** Confocal time lapse microscopy of *C. neoformans* exocytosis by wild type *D. discoideum* strain Ax2. Representative time lapse from three independent experiments. Images were captured every 5 s. Arrow indicates exocytosed cryptococcal cell. Inset box is a magnification of exocytosed cryptococcal cell demonstrating absence of V-ATPase prior to exocytosis. Scale bars 10 μm.

During normal transit, phagosomes rapidly accumulate the V-ATPase and acidify within 2–3 min of engulfment; the V-ATPase is subsequently retained for ~45 min to allow digestion before retrieval and phagolysosomal neutralization prior to constitutive exocytosis (Clarke et al., 2002, 2010). Using cells expressing GFP-fused to the VatM subunit of the V-ATPase, we monitored recruitment to phagosomes following engulfment of cryptococci. V-ATPase was present, on average, within 114 ± 44 s (Figure 2A, S.D., *n* = 6) of phagocytosis consistent with published data for inert phagosomes (Clarke et al., 2002; Buckley et al., 2016). To test if V-ATPase recruitment was maintained, we also measured the proportion of *C. neoformans*-containing phagosomes positive for GFP-VatM after 20 min incubation of cryptococci with amoebae—before any post-lysosomal transitions should have occurred (Figure 1F). V-ATPase was clearly visible on 90.0% of phagosomes (68/76 from three independent experiments). However, when we looked after 1 h, we were able to observe recycling of the V-ATPase from live (budding) *C. neoformans*-containing phagosomes prior to exocytosis (Figure 2B). Both phagosomal transit time and V-ATPase dynamics are therefore unaffected by pathogenic *C. neoformans* indicating that the normal mode of release is through canonical constitutive exocytosis.

### *C. neoformans* can escape from amoebae by both WASH-dependent and -independent mechanisms

To test the hypothesis that Cryptococci-containing phagosomes are normally expelled through the constitutive exocytosis pathway, we investigated exocytosis in *WASH*-null cells. In *D. discoideum*, WASH is essential for V-ATPase recycling and constitutive exocytosis, allowing us to specifically genetically ablate this pathway (Carnell et al., 2011).

**Figure 3:**
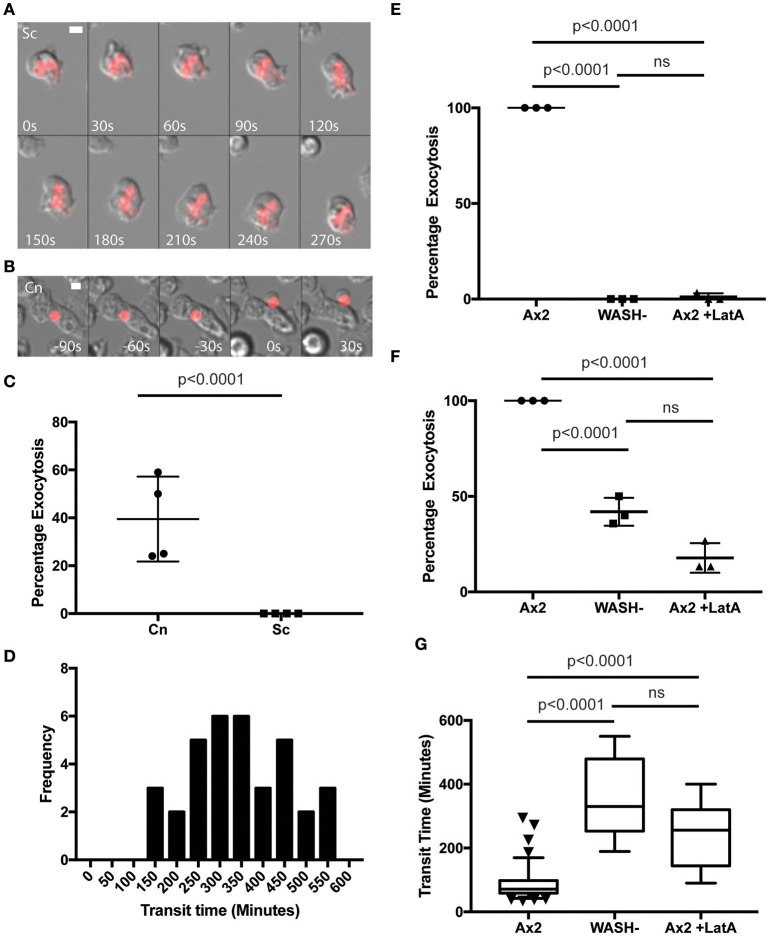
Exocytosis of *Cryptococcus neoformans* from *Dictyostelium* is dependent on WASH and the actin cytoskeleton. **(A)** Heat killed *S. cerevisiae* are not exocytosed from WASH null *D. discoideum*. Example from 12 h time lapse imaging of heat killed *S. cerevisiae* in WASH null *D. discoideum* representative of 60 phagosomes containing heat killed *S. cerevisiae* from three independent experiments (images start at 500 min after phagocytosis). **(B)** *C. neoformans* are exocytosed from WASH null *D. discoideum*. Example from 12 h time lapse imaging representative of 62 phagosomes containing *C. neoformans* from three independent experiments (images start at 200 min after phagocytosis). Scale bars 5 μm. **(C)** Quantification of the percentage of exocytosis of *C. neoformans* and heat killed *S. cerevisiae* from WASH null *D. discoideum* from three independent 12 h time lapses. **(D)** Frequency histogram of combined 35 transit times measured from three independent 12 h time lapses. **(E)** Exocytosis of heat killed *S. cerevisiae* from wild type Ax2 but not WASH null or latrunculin A treated Ax2 *D. discoideum*. Quantification of the percentage of exocytosis from three independent 12 h time lapses. Total of 60 phagosomes were analyzed from each condition. *P*-values are Fishers test. **(F)** Exocytosis of *C. neoformans* from wild type Ax2, WASH null, and latrunculin A treated *D. discoideum*. Quantification of the percentage of exocytosis from three independent 12 h time lapses. Total of 60 phagosomes were analyzed from each condition. *P*-values are Fishers test. **(G)** Transit times of *C. neoformans* through WASH null and latrunculin A treated Ax2 *D. discoideum* are not significantly different. *P*-values are Mann-Whitney test.

In agreement with previous studies we found that phagosomes containing heat killed *S. cerevisiae* were never released from *WASH*-null amoebae within our 12 h period of observation (Figure 3A). In contrast, phagosomes containing *C. neoformans* were still exocytosed but with significantly altered dynamics (Figure 3B). Whilst >90% of *C. neoformans*-containing phagosomes are exocytosed within 2 h in wild type amoebae (Figure 1F), release from *WASH*-null cells was much more variable with between 20 and 60% escaping over 12 h (Figure 3C). Cryptococcus-containing phagosome transit was much slower through *WASH*-null cells with very little overlap with the exocytosis from wild-type amoebae (ca. Figures 1F, 3C) and was much less synchronous, appearing to occur stochastically any time from 3 to >10 h (Figure 3D). Notably, both this variation and timing is comparable to that reported for vomocytosis of cryptococci from animal cells (Johnston and May, 2010).

The actin cytoskeleton is a negative regulator of vomocytosis in mammals, but essential for constitutive phagosome exocytosis in *D. discoideum* (Ma et al., 2006; Carnell et al., 2011). Therefore, we predicted that inhibition of actin polymerization would copy the phenotype of *WASH*-null *Dictyostelium* for both heat killed *S. cerevisiae* and *C. neoformans* containing phagosomes. When we measured the percentage exocytosis of heat killed *S. cerevisiae* containing phagosomes we found that while 100% of phagosomes were exocytosed by untreated amoebae we only observed a single exocytosis event out of 90 phagosomes analyzed over 12 h when actin polymerization was blocked by latrunculin A treatment after phagocytosis (Figure 3E). In contrast, ~20% of *C. neoformans*-containing phagosomes in latrunculin A-treated amoebae were released over the same period (Figure 3F). Transit times were again significantly longer than with untreated amoebae, and were indistinguishable to the phenotype observed with *WASH*-null amoebae (Figure 3G). Thus, whilst cryptococci are normally released by constitutive exocytosis from *D. discoideum*, they can also escape by a mechanistically different route upon either pharmacological or genetic blockade of the constitutive pathway, highly reminiscent of vomocytosis.

### *C. neoformans* mutants *cap59* and *plb1* do not exhibit defects in vomocytosis in amoebae

**Figure 4:**
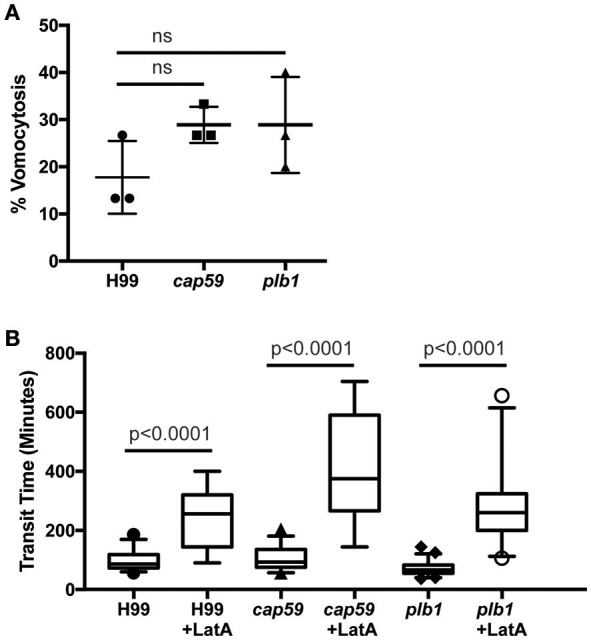
Exocytosis and vomocytosis of *C. neoformans cap59* and *plb1* mutants are indistinguishable from wild type H99. **(A)** Quantification of the percentage of non-constitutive exocytosis of *C. neoformans* mutants from wild-type Ax2 *D. discoideum* treated with latrunculin A from three independent 12 h time lapses. Total of 45 phagosomes were analyzed for wild type H99, 90 for *cap59* and 90 for *plb1*. *P*-values are Fisher's exact test. **(B)** Transit times of *C. neoformans* H99 wild type, mutant *cap59* and *plb1*. Phagosomes analyzed the same as **(A)**. *P*-values for between cryptococcal strain significance tests are non-significant in all cases. *P*-values are Mann-Whitney test.

We next tested whether cryptococcal virulence factors that affect vomocytosis from mammalian cells play conserved roles in egress from *D. discoideum*. *C. neoformans* mutants with defects in polysaccharide capsule formation or deletion of the phospholipase PLB1 both exhibit reduced rates of vomocytosis and pathogenicity in animal cells (Cox et al., 2001; Noverr et al., 2003; Chayakulkeeree et al., 2011; Evans et al., 2015). However, when we measured the rates of release of acapsular *cap59* and *plb1* mutant strains from wild-type *D. discoideum* we found that release of both mutants was unaffected: there were no significant differences in either frequency or transit time compared to the parental *C. neoformans* strain (H99) both with and without blockage of the constitutive pathway with latrunculin A (Figures 4A,B). This was an intriguing finding, suggesting differences in the signaling pathways to vomocytosis between amoebae and macrophages but a conservation of molecular mechanism.

### In the absence of constitutive exocytosis *C. neoformans* can persist and grow intracellularly

Constitutive exocytosis of indigestible phagosomal material is critical for organisms that rely on phagocytosis for nutrition. Furthermore, amoebae may ingest microbes such as cryptococci that are able to actively resist phagosomal killing. Therefore, we investigated if, in the absence of constitutive exocytosis, cryptococci posed a greater threat to amoebae.

**Figure 5:**
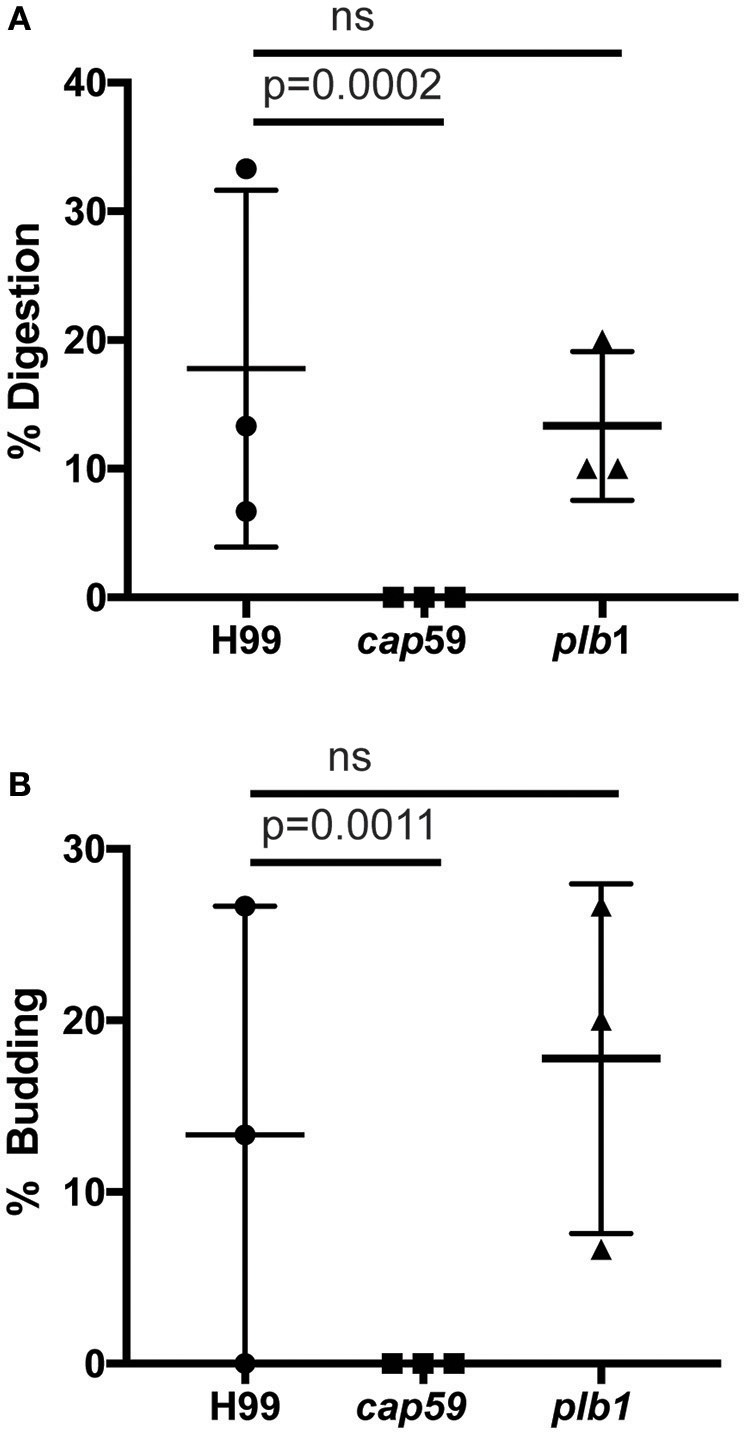
Outcomes of interaction between cryptococcal cells and amoebae with inhibition of constitutive exocytosis. Quantification of outcome from three independent 12 h time lapses. Numbers of phagosomes analyzed the same as Figure 4A. **(A)** Percentage of fungal cells digested by amoebae over 12 h. **(B)** Percentage of fungal cells that budded while intracellular in amoebae over 12 h. *P*-values are Fisher's exact test.

We observed no cell lysis of either wild-type or *WASH*-null cells infected with *C. neoformans* as indicated by the continued motility of the amoebae throughout the phagocytic cycle and after fungi egress. Low levels of amoeba lysis were observed upon latrunculin A treatment, most likely due to the severe effects of complete actin depolymerization. Killing and digestion of yeast is indicated by the transformation of the phagosome to a granular and irregular shape, whereas growth can be inferred from yeast budding. When constitutive exocytosis was blocked with latrunculin A on average, more than 80% of cryptococci persisted within *Dictyostelium* phagosomes without signs of death and digestion, with 13% actively budding over the 12 h of the experiment (Figures 5A,B). In this respect the *plb1* mutant behaved similarly to wild type cryptococci. The acapsular strain *cap59* was phagocytosed twice as efficiently as the wild type cryptococcus strain (11 internalized *cap59* cryptococci per 100 amoeba vs. 5.3 cryptococci per 100 amoeba, 300 amoeba analyzed from three independent experiments). However, the acapsular *cap59* strain appeared to be growth-arrested within *D. discoideum* phagosomes, as budding was never observed (0/90 phagosomes, *P* = 0.0011 compared to H99, Fishers test; Figure 5B). Whilst this is consistent with other studies (Feldmesser et al., 2000; Steenbergen et al., 2001) surprisingly, we also never observed the collapse of *cap59*-containing phagosomes within the 12 h indicating yeast death. This implies that either acapsular cells survive under growth arrest, or that death of *cap59* cells occurs over a longer period than we are able to observe in this assay. Nonetheless, in the absence of constitutive exocytosis, yeast are able to replicate intracellularly within the retained phagosomes.

## Discussion

In this work we have investigated the interactions between the environmental yeast *C. neoformans* and an environmental amoeba, *D. discoideum*. As *C. neoformans* can evade human immune cells and cause opportunistic infection in immunocompromised individuals we sought to test the hypothesis that the yeast virulence mechanisms had evolved to protect against amoebae in the environment.

In agreement with previous reports (Steenbergen et al., 2003), we found that *C. neoformans* were almost completely impervious to predation by *D. discoideum*; we consistently observed lower rates of *C. neoformans* phagocytosis compared to other particles, and even when the yeast were engulfed the amoebae were unable to kill them. This is similar to the interactions with mammalian macrophages, indicating that *C. neoformans* employs similar phagocyte evasion strategies. Chief amongst these is the characteristic cryptococcal capsule which is highly anti-phagocytic and also provides protection from oxidative attack. Consistent with this both we, and others found capsule deficient yeasts were unable to grow within amoebae (Steenbergen et al., 2001, 2003) and we found that the acapsular strain was taken up twice as frequently as the wild type.

Whilst *C. neoformans* typically persist and proliferate in macrophages for many hours before escaping by vomocytosis, we found they passed through *D. discoideum* via an apparently normal phagosomal transit in just ~80 min. This short time period provides limited opportunity for intracellular growth of fungal cells or the lysis of amoebae. Whilst others have shown that *C. neoformans* are able to grow (and therefore extract nutrients) when co-incubated with amoebae over several days (Steenbergen et al., 2001, 2003). The relatively short amount of time spent inside the amoeba implies that much of this growth is extracellular and intracellular growth has not been directly demonstrated by high resolution time lapse imaging (Steinman et al., 1976; Derengowski et al., 2013; Fu and Casadevall, 2018). We were unable to identify intracellular proliferation in the absence of the constitutive exocytosis inhibition. It therefore seems unlikely that *C. neoformans* has successfully evolved mechanisms to support intracellular replication within amoebae that exhibit rapid phagosomal transit, as demonstrated for *D. discoideum, Amoeba proteus, Entamoeba histolytica* and *A. castellanii* (Weisman and Korn, 1967; Ravdin et al., 1988; Christofidou-Solomidou and Stockem, 1992; Clarke et al., 2010).

Although *D. discoideum* are unable to kill phagocytosed *C. neoformans* the transit time is identical to that of heat-killed non-pathogenic yeast. This suggests that there is no major subversion of normal phagosome maturation. Consistent with this we find that the V-ATPase is both recruited, and retrieved from phagosomes with normal dynamics. This is in contrast to a recent study in macrophages, demonstrating that *C. neoformans* is able to disrupt phagosome maturation to inhibit acidification and proteolysis to permit intracellular proliferation (Smith et al., 2015). As the crypotococcal-containing phagosome in macrophages is permeabilized shortly after phagocytosis by macrophages (Tucker and Casadevall, 2002) V-ATPase delivery may be intact in both systems, but ineffective due to proton leakage.

Surprisingly, although *C. neoformans* are released alive from *D. discoideum* by canonical, WASH-dependent constitutive exocytosis, we found they were still expelled when the constitutive pathway was blocked. This second pathway strongly resembles vomocytosis from macrophages, being non-lytic, stochastic and inhibited by actin (Alvarez and Casadevall, 2006; Ma et al., 2006; Johnston and May, 2010). Vomocytosis remains mechanistically poorly understood and defined, but it seems highly likely that the WASH-independent egress of *C. neoformans* from *D. discoideum* is an analogous process.

Whilst laboratory strains have mutations that facilitate macropinocytosis and phagocytosis of large particles, wild-type isolates of *D. discoideum* are bacterivores and cannot engulf yeasts (Bloomfield et al., 2015). Therefore although *D. discoideum* provides a genetically tractable model for amoebae in general, it is highly unlikely to be an environmental host for cryptococci. The high phagocytic throughput of amoebae necessitates a mechanism to dispose of indigestible material, and unlike macrophages, there is no advantage in retaining phagosomes indefinitely to restrict an inflammatory response. As constitutive exocytosis appears to be sufficient for successful escape of *C. neoformans* on its own, the evolutionary drivers of a secondary, redundant escape mechanism are unclear. Whether other, more environmentally-relevant amoeba behave differently, or another environmental interaction altogether selects for this virulence trait requires further study.

Whatever the evolutionary basis, we have shown that vomocytosis-like egress is a mechanistically distinct process from constitutive exocytosis and is conserved in hosts from *D. discoideum* to man. The genetic tractability of *Dictyostelium* amoebae present an unparalleled opportunity to study the molecular cell biology of host cryptococcal interactions, and the differences in the environmental niche of *C. neoformans* and infection of humans.

## Author contributions

RW, AA, CW, and CB performed experiments and data analysis. Initial concept, funding, experimental design were undertaken by JK and SJ. All authors prepared and edited the manuscript.

### Conflict of interest statement

The authors declare that the research was conducted in the absence of any commercial or financial relationships that could be construed as a potential conflict of interest.
